# Humoral and Cellular Immune Responses of COVID-19 vaccines against SARS-Cov-2 Omicron variant: a systemic review

**DOI:** 10.7150/ijbs.73583

**Published:** 2022-07-11

**Authors:** Zhonghao Chen, Ying Zhang, Meng Wang, Md Sahidul Islam, Ping Liao, Yuanjia Hu, Xin Chen

**Affiliations:** State Key Laboratory of Quality Research in Chinese Medicine, Institute of Chinese Medical Sciences, University of Macau, Macau SAR 999078, China

**Keywords:** SARS-COV-2, Omicron variant, vaccine, booster dose, humoral immune response, cellular immune response

## Abstract

Severe Acute Respiratory Syndrome Coronavirus 2 (SARS-CoV-2) has undergone multiple mutations since its emergence, and its latest variant, Omicron (B.1.1.529), is the most contagious variant of concern (VOC) which poses a major and imminent threat to public health. Since firstly reported by World Health Organization (WHO) in November 2021, Omicron variant has been spreading rapidly and has become the dominant variant in many countries worldwide. Omicron is the most mutated variant so far, containing 60 mutations in its genome, including 37 mutations in the S-protein. Since all current COVID-19 vaccines in use were developed based on ancestral SARS-CoV-2 strains, whether they are protective against Omicron is a critical question which has been the center of study currently. In this article, we systemically reviewed the studies regarding the effectiveness of 2- or 3-dose vaccines delivered in either homologous or heterologous manner. The humoral and cellular immune responses elicited by various vaccine regimens to protect against Omicron variant are discussed. Current understanding of the molecular basis underlying immune escape of Omicron was also analyzed. These studies indicate that two doses of vaccination are insufficient to elicit neutralizing antibody responses against Omicron variant. Nevertheless, Omicron-specific humoral immune responses can be enhanced by booster dose of almost all type vaccines in certain degree, and heterologous vaccination strategy may represent a better choice than homogenous regimens. Intriguingly, results of studies indicate that all current vaccines are still able to elicit robust T cell response against Omicron. Future focus should be the development of Omicron variant vaccine, which may induce potent humoral as well as cellular immune responses simultaneously against all known variants of the SARS-CoV-2 virus.

## 1. Introduction

During the COVID-19 pandemic, Severe Acute Respiratory Syndrome Coronavirus 2 (SARS-CoV-2) has evolved more than 1,500 different Pango lineages in more than 270 million cases reported 1. The new coronavirus already had four major variant of concerns (VOCs), namely Alpha, Beta, Gamma, and Delta. On 24th November 2021, a new variant (B.1.1.529), named Omicron, was reported to WHO by South Africa. On 26th November, the WHO defined it as the fifth VOC. Omicron variant infection was found in 128 countries and territories by 4th January 2022 2. Studies have shown that Omicron has an even farther antigenic distance from the ancestor virus, as compared to that of Beta and Delta variants. As a result, the protective efficacy of previous vaccines against Omicron was markedly reduced 3.

As the most severely mutated strain, there are 18,261 mutations in various Omicron isolates, in which 97% of them are in coding regions 4. The entry of the SARS-CoV-2 virus into the host cell is through the Spike-protein(S-protein) which acts on the angiotensin converting enzyme II (ACE2) receptor of the host cells 5. There are 30 mutations in the S-protein of Omicron, most of which are in the receptor binding domain (RBD) region 6. The RBD of Wuhan-Hu-1 has 1,273 amino acids, in contrast, the RBD of Delta and Omicron variants has 1,271 and 1,270 amino acids, respectively 7. Several major previously appeared mutations and some novel mutations were found in Omicron variants, which increase the risk of infection and possibly resistance to existing vaccines 8.

Vaccination programs have been initiated in many countries, and in some countries, there is wider coverage. Vaccination elicits neutralizing antibodies against key viral proteins, such as S-protein, has been effectively slowing down the spread of the virus and reducing the rate of severe morbidity and mortality 9. In spite of this, the reduced sensitivity of neutralizing antibodies to key viral proteins of the variants, including SARS-CoV-2 Omicron, resulted in an increase in the risk of immune escape and a reduction of the effectiveness of the vaccine 8, 10. Currently, understanding the effectiveness of various vaccine regimens against Omicron variant has become the focus of research. Although remained incompletely understood, the humoral and cellular immune responses elicited by current vaccines to Omicron have been intensively studied. In this article, we systemically reviewed the latest progress in this fast-evolving field.

## 2. Major SARS-CoV-2 VOCs and the protective efficacy of vaccines

**Alpha (B.1.1.7).** At the end of December 2020 in the United Kingdom, a new SARS-CoV-2 virus was discovered. The Alpha strain, also known as the B.1.1.7 strain, contains mutations such as 69/70/144del, N501Y, A570D, P681H, T716I, S982A, and D1118H in the spike protein. Among them, the N501Y mutation can lead to enhanced viral adhesion, making it easier to enter host cells 11.

**Beta (B.1.315).** Beta strain is the main strain for secondary outbreaks of COVID-19. It was found in South Africa in October 2020 12. The spike protein of Beta strain contains 9 mutation sites, which are L18F, D80A, D215G, R246I, K417N, E484K, N501Y, D614G, and A701V 12. Three mutations, e.g., K417N, E484K, and N501Y, are in the RBD which enhanced the binding affinity of B.1.315 to ACE2 receptor 13.

**Gamma (P.1).** Gamma strain was firstly spotted in Brazil in January 2021^14^. The spike protein of Gamma strain contains 11 mutations, including L18F, T20N, P26S, D138Y, R190S, H655Y, T1027I, D614G, K417T, E484K, and N501Y 15. Among them, three mutations (L18F, K417T and E484K) are located in the RBD region which may improve the binding affinity of P.1 strain to ACE2 15.

**Delta (B.1.617.2).** Delta strain is the fourth VOC which was initially found in India in December 2020. The spike protein of Delta strain contains 10 mutations (T19R, G142D, 156/157del, R158G, L452R, T478K, D614G, P681R and D950N) 16.

**Omicron (B.1.1.529).** On 26th November 2021, the fifth VOC was identified and named as Omicron. The variant has rapidly spread to many regions throughout Asia, Africa, Europe, and North America 17. This strain is currently the most severely variant with many mutation sites in its spike protein species, including 69-70/142-144/211del A67V, T95I, Y145D, L212I, ins214EPE, T547K, D614G, H655Y, N679K, P681H, N764K, D796Y, N856K, Q954H, N969K, and L981F 18. Omicron has further evolved into three subvariants, namely Omicron BA.1 (B.1.1.529.1), BA.2 (B.1.1.529.2), and BA.3 (B.1.1.529.3). Among them, BA.2 shares 32 same mutations with BA.1, while possessing distinct 28 mutations. BA.2 was found to be able to re-infect patients who were already infected with BA.1^19^, suggesting that BA.2 may become the protagonist of the next wave of epidemic after the Omicron BA.1 pandemic 19.

### Protective Effect of Vaccines against various VOCs of SARS-COV-2

To date, four main categories of SARS-CoV-2 vaccines, e.g. mRNA, adenoviral vector, inactivated, and recombinant subunit vaccines, have been developed and approved for use. As of 15th March, 2022, a total of 216 vaccines are in development and 92 of them have entered the stage of human trials 20. Due to the severity of the epidemic beyond the ability to control, WHO has included 10 vaccines on the emergency use listing (EUL), as summarized in Table 1. Studies showed that the most of vaccines are effective in protection against Alpha variant 21-24. However, their efficacy against Beta variant was markedly decreased 22, 23. Effect of vaccines against Gamma variant was further reduced 25, while the immune escape rate of Delta variant remains the same as that of Gamma variant 26.

SARS-CoV-2 Omicron is highly transmissible even among fully vaccinated individuals 27, 28, suggesting that this variant has the capacity to evade protective immune responses elicited by current vaccines. Compared to the original wild-type (WT) strain of SARS-CoV-2, Omicron variant has 60 amino acid mutations, 37 of which are in the S-protein, and these mutations in the S-protein RBD which binds to the ACE2 receptor will change binding epitopes to many current antibodies 29. G446S is a key antibody escape site. By altering the local conformation of the binding interface, G664S provides greater resistance to a major class of antibodies binding to the right shoulder of the RBD 30. Right shoulder represents a region of the RBD surface named by a conventional nomenclature compared with human torso 31. The structural plasticity at the RBD-ACE2 interface promotes the accumulation of a large number of RBD mutations of Omicron variant which lead to the reduction of the efficacy of neutralizing antibody 32, 33.

## 3. Vaccine-induced humoral immune responses against Omicron variant

S-protein, a homotrimeric protein, is located on the surface of the virus membrane 34. Each of its monomers is composed of two domains, S1 and S2. S1 is responsible for receptor binding and S2 is responsible for membrane binding. S1 consists of an RBD and an N-terminal domain (NTD), RBD directly binds to ACE2 and NTD is involved in the regulation of S-protein activation and influences the RBD binding process 34. The traditional neutralizing antibodies are to block the binding site between RBD and ACE2, many vaccines are developed for this, such as BNT162b2 and ZF2001 35. However, recent studies found that anti-NTD also can inhibit the RBD binding processes 34. Among 86 potent neutralizing antibodies identified in peripheral blood mononuclear cells from vaccinees, the diversity of anti-RBD antibodies is much higher than anti-NTD antibodies 36. RBD and NTD have accumulated a high number of mutations in SARS-CoV-2 variants. These mutations have direct implications on virus infection rates through the higher affinity of RBD for the ACE2 receptor and escape of neutralizing antibodies 37. Therefore, variants of SARS-CoV-2 can escape neutralization by maintaining these mutations, which results in the failure of vaccination. It is worth mentioning that SARS-CoV-2 RBD-specific memory B cell clones accumulated over time, and the pool of memory B cells may provide long-term protection 38. Clonal relationships indicated that at least some of these somatic mutations lead to part of cross-reactivity of memory B cells 39. Memory B cells play the central role in humoral immune response; they are generated in prolonged germinal center reactions 40. These memory B cells actually increased between 1 month and 8 months after infection, accompanied with affinity maturation of antibodies. The kinetics of memory B cells in humans after an initial acute viral infection is poorly understood, and further study is needed 41. A study on vaccinated individuals revealed that mRNA vaccines induced variant-specific memory B cells maintained for at least 6 months and such B cells could cross-bind with Alpha, Beta and Delta variants 39. To date, the response of memory B cells to Omicron remains unclear.

In the following sections, we will discuss the humoral immune responses elicited by two or three doses of different types of vaccines against SARS-CoV-2 Omicron variants, followed by the analysis of evidence showing the enhanced efficacy of booster dose. The humoral immune responses elicited by heterologous versus homologous vaccination strategies are also compared.

### Diminished humoral immune responses against Omicron by 2-dose of vaccines

**mRNA Vaccines (BNT162b2 and mRNA-1273).** There is compelling evidence that the efficacy of two doses of mRNA vaccines in neutralization of Omicron variant was markedly reduced. In the individuals vaccinated with two-dose of BNT162b or mRNA-1273, the titers of neutralizing antibodies against Omicron were 23~122 folds lower than antibodies against WT, ancestral strain or mutant strains (such as USA-WA1/2020, Victoria and D614G strains) 42-52. However, the binding capacity of antibodies elicited by 2-does mRNA vaccines was preserved to some degree, as the difference in binding affinity of antibodies to RBD of Omicron and that of WT strain was minor, with 2.9-fold reduction in individuals vaccinated with 2-dose mRNA-1273 and 1.5-fold reduction after boosted with BNT162b2 as compared to WT 44. Due to the differences in the design of study, experimental condition and sample sources, the results between the above studies may not be comparable.

**Adenoviral vector vaccines (ChAdOx1 or AZD1222, Covishield and Ad26.COV2.S).** It was shown that the effectiveness of two doses of ChAdOx1was reduced over time, with a limited protection against symptomatic disease caused by the Omicron variant for 20 to 24 weeks after the second vaccination 53. A study shows that, in 21 out of 22 individuals vaccinated with two doses of ChAdOx1, the serum neutralizing antibody titers against Omicron dropped below detectable thresholds 50. Similarly, another study found that none of the individuals received 2-dose of ChAdOx1 had cross-neutralization of the Omicron variant 54. As for Covishield, a study found that the neutralizing antibody titers to Omicron variant were reduced by 23.15-fold in individuals vaccinated with two doses 55. As for Ad26.COV2.S, Omicron neutralizing antibody titers were reduced by 17-fold to WT, with low level GMNT in individuals either vaccinated recently (42 IU/mL) or vaccinated six months later (33 IU/mL) was found in individuals received 1 dose vaccine 42. Therefore, the humoral immune responses against Omicron elicited by adenoviral vector vaccines appear to be minimal.

**Inactivated vaccines (BBIBP-CorV, CoronaVac and COVAXIN)**. It was shown that none of the individuals fully vaccinated with CoronaVac had detectable neutralizing antibodies against Omicron variant 56. Another study found about 6.4-fold decrease of GMT against Omicron variant from recipients who received 2-dose of CoronaVac as compared to GMT against WT strain. Again, none of them was above 50% threshold of protection 57. Plasma collected on day 14 after a second dose of CoronaVac showed a 12.5-fold reduction in average in neutralizing Omicron variants as compared to WT strain 58. Another study showed that after 30 days of second dose, CoronaVac immunization generated detectable neutralizing antibodies against Omicron in only 17% of individuals, which dropped to 10% after 60 days 59. As for BBIBP-CorV, it was shown that GMT against Omicron was still above the low limit after 4~8 months 60. As compared with WT strain, a 1.52-fold reduction in levels of neutralizing antibody against the Omicron variant was observed in recipients who received 2-dose of BBIBP-CorV 61. As for COVAXIN, one study found that the levels of neutralizing antibody against Omicron in individuals vaccinated with 2 doses were reduced 8.67~12.49-fold, as compared to B.1 strain. However, even 6 months after initial vaccination, the anti-S1-RBD and nucleocapsid IgG neutralizing antibodies could still be detected 62, 63.

Taken together, the above studies indicate that the titers of neutralizing antibodies against Omicron elicited by 2-dose of all current vaccines are markedly reduced, as compared to those against ancestral strain of SARS-CoV-2 (summarized in Table 2). These data indicate that 2-dose of vaccination are insufficient to generate satisfactory levels of protection against infection with Omicron and mild disease. In fact, such reduction appears to be more profound in individuals who received adenovirus vaccine, as compared to those who received mRNA vaccines 64. Neutralizing antibodies against Omicron elicited by standard two inoculations of inactivated vaccines is often low, indicative of the limited capacity of this type of vaccine in the induction of humoral immune responses against Omicron 57, 61. Nevertheless, although the full vaccination frequently fails to show a strong neutralizing antibody activity against Omicron variant, they still have clear public health benefit such as reduction of infectious viral load and transmission risk 65.

### Humoral immune response against Omicron was enhanced by booster dose of vaccines

Since humoral immune responses against Omicron variant elicited by all current vaccines were markedly reduced, many countries and regions are calling for an additional booster vaccination six months after the second dose. It was shown that the booster dose resulted in a significant improvement in protection against mild diseases 53. A real-world study that analyzed about 62,000 vaccinated individuals in UK revealed that participants who had received 2 or 3 doses of vaccines had a lower risk of hospitalization during Omicron infection. And individuals who had received 3 doses of the vaccine have a marked reduction on the duration of Omicron symptoms (2-dose: 8.3 days, 3-dose: 4.4 days ) 66. The importance of booster dose is evident, since it can elicit higher level of neutralizing antibody against the Omicron 47.

It was shown that over 90% of individuals retained neutralizing activity against the Omicron after booster dose of mRNA vaccines 52. As compared to 2-dose of mRNA vaccines, booster dose could enhance the titers of neutralizing antibodies against Omicron variant about 20~30 times 51, 67. For example, individuals vaccinated with 3-dose of BNT162b2 demonstrated a minimal reduction of neutralizing antibody against Omicron (4-fold decrease) 48. Another study also showed that the booster dose of BNT162b2 resulted in a potent neutralizing activity against Omicron (ED_50_= 722) in individuals with a low neutralizing activity after two doses of BNT162b2 (ED_50_ < 30) 68. Similarly, after booster vaccination, the effective serum neutralizing activity in individuals received BNT162b2 was increased from 37% to 100% 49. Other studies based on pseudo virus neutralization experiments also reported the similar result, with 16~31.8-fold increase in the neutralizing activity 42, 45, 46, 57. The neutralizing activity against Omicron remained robust even 4 months after booster dose vaccination with BNT162b2 69.

The effect of booster dose of mRNA-1273 was also studied. For example, it has been reported that boosting with another dose of mRNA-1273 resulted in a 19~32.3-fold increase in cross-neutralization of the Omicron variant 42 70. One study estimated that the effectiveness of 3-dose of BNT162b2 and mRNA-1273 were 66% and 69%, respectively 71. Another study showed that the reduction of neutralizing activities against Omicron in individuals received booster dose of BNT162b2 or mRNA-1273 was 7.5-fold and 16.7-fold respectively, as compared to their effect on USA-WA1/2020 44. Further investigation is needed to determine if such differences between two major mRNA vaccines could be indicator of better booster dose of vaccine.

The booster dose of inactivated vaccines could also enhance the production of neutralizing antibodies against Omicron variant. For example, it was reported that BBIBP-CorV vaccine booster resulted in a 1.5~5-fold increase in the neutralizing antibodies against Omicron, as compared to that elicited by 2-dose of the same vaccine 60 61 72. Similarly, in recipients vaccinated with COVAXIN, the booster dose of same vaccine increased the levels of neutralizing antibodies against Omicron variant by 18.53-fold 62. However, one study failed to observe the increase of neutralizing activity against Omicron by booster dose of CoronaVac 57.

Taken together, there is strong evidence that the booster dose of the most of current vaccines can markedly enhance the humoral immune responses against Omicron variant, in terms of GMT of neutralizing antibody in the serum (summarized in table 3). There is evidence that mRNA vaccines as booster dose can elicit markedly higher levels of neutralizing antibodies than inactivated vaccines (GMT for mRNA vaccines: 93- 3179, GMT for inactivated vaccines:7.6- 84) (summarized in table 3). It was shown that antibody diversity can be increased through repeated antigen exposure which may help neutralizing SARS-CoV-2 variants 73. Indeed, the booster dose is able to improve the breadth of humoral response and cross-reactivity to mutated SARS-CoV-2 variants such as Omicron, in addition to increasing the neutralizing antibody titers 42. The difference in cross-reactivity between two doses and third dose may be due to emerging neutralizing antibodies that can target new conserved epitopes on S-protein or increasing the affinity of existing neutralizing antibodies, rendering them less sensitive to mutations in their target epitopes, or both 42. Thus, the booster dose of the most of current vaccines is necessary and can provide a stronger protection against Omicron variant, as compared to 2-dose vaccines. Nevertheless, although booster dose substantially increases the protection against Omicron, the immune responses triggered by it can wane over time 53. To date, the effect of adenoviral vector vaccines as primary and booster vaccination on the titers of neutralizing antibody against Omicron has not been reported yet, since U.S. FDA just approved Ad26.COV2.S as booster dose on 21st October 2021.

### Heterologous vaccination regimens improve humoral immune response against Omicron variant

In addition to the aforementioned booster dose with the same vaccine (homologous vaccination regimens), vaccines of different technical platforms, e.g. heterologous vaccination regimens, have been used in the priming dose and booster dose, in the hope of enhancing the production of neutralizing antibodies against Omicron variant. In the current vaccination program, almost all reported adenovirus vector vaccines were used as a priming vaccination and boosted with mRNA vaccines 42, 74. It was found that GMT of neutralizing antibody against Omicron variant in individuals received 2-dose ChAdOx1 and a booster dose of BNT162b2 was comparable with GMT in individuals vaccinated with 3 doses of BNT162b2 74. It is worth noting that antibody binding doesn't represent the whole function of antibody, since neutralization is more important. A pseudo virus neutralization assay-based study indicated that Ad26.COV2.S vaccines boosted with mRNA-1273 also showed a substantially enhanced neutralizing activity against Omicron variant 42. One study using VISION Network analysis found that one dose of Ad26.COV2.S vaccine plus one dose of mRNA vaccine yielded better protection than two doses of Ad26.COV2.S, and the efficacy of heterologous regimen was comparable to that of 3 doses of mRNA vaccines 75. Further investigation is needed to verify the observation that booster dose of mRNA vaccine can improve the efficacy of adenovirus vaccine. In India, COVAXIN was used as booster dose in individuals received Covishield as prime dose and found that IgG titer increased 1.65- and 1.18-fold to those vaccinated with Covishield or COVAXIN only, respectively 55, suggesting that inactivated vaccine may also enhance humoral immune responses induced by adenovirus vaccine to certain degree.

Homologous booster strategy with inactivated vaccinees can also enhance the neutralizing effect against Omicron variant, but less potent than mRNA vaccines 57. Thus, mRNA vaccine was proposed as booster dose for individuals received priming dose of inactivated vaccine. For example, it was shown that the individuals primarily vaccinated with 2-dose CoronaVac and received booster dose of BNT162b2 exhibited significantly elevated levels of Omicron-specific antibodies which was 1.4-fold higher than in individuals who received 2-dose of mRNA vaccines 56. Another study using a viral microneutralization assay with SARS-CoV-2 Omicron live virus found that the mean titers of recipients vaccinated with 2-dose of CoronaVac and 1-dose BNT162b2 increased 27.5-fold, as compared to 2-dose of CoronaVac 59. This improvement may be caused by the fact that beta-coronaviruses including SARS-CoV-2 variants share a conserved site in S-protein, and BNT162b2 used S-protein mRNA from the original Wuhan SARS-CoV-2 isolate 59. In addition, ZF2001, a recombinant subunit vaccine which was developed with recombinant DNA technology using the RBD of the S-protein as antigen, as booster dose brought about 10-fold increase in GMT of neutralizing antibody against Omicron in the individuals received BBIBP-CorV as priming dose 60. Another study that used the same vaccination regimens also found that booster dose elicited the neutralizing antibodies that targeted some conserved sites in RBD or NTD 61. Moreover, a large-scale real-world data from Brazil showed that the effectiveness of 2-dose of CoronaVac was 55.0% against infection and 82.1% against severe outcomes during the period of 14~30 days after vaccination, and such effect was decreased to 34.7% and 72.5% after 180 days post vaccination, respectively. However, administration of booster dose of BNT162b2 after 6 months could markedly enhance the effectiveness to 92.7% against infection and 97.3% against severe outcomes in the following 14~30 days, although the elderly was less responsive to the booster dose 76.

In summary, there are strong evidence that the humoral immune responses against Omicron variant could be markedly increased by heterologous vaccination regimens (summarized in Table 3). More specifically, booster dose of mRNA vaccines can elicit high levels of neutralizing antibodies against Omicron variant in individuals received adenovirus vaccines or inactivated vaccines as priming dose. Using recombinant subunit vaccine to boost inactivated vaccines can also enhance the neutralizing activity against Omicron variant. Adenovirus vaccines may also be useful as booster dose to enhance the efficacy of inactivated vaccines. The results from recent studies strongly suggest that the heterologous regimens including different types of vaccines developed with distinct technology platform can induce stronger humoral immune responses to emerging variants including Omicron. Importantly, heterologous regimens containing at least one dose of mRNA vaccine markedly enhance the binding and neutralizing activities of elicited antibodies against Omicron variant, while its safety is comparable to that of homologous regimens, as shown by a UK study 77.

A recent UK study indicated that the effectiveness of heterologous vaccine regimens against hospitalization dropped from 92% to 83% just 10 weeks after a third dose 78, suggesting that the protection provided by a booster dose could also wane over time. Therefore, the efficacy of the fourth dose of vaccine was investigated. For example, two studies from China and Israel showed that the relatively high levels of cross-neutralizing antibodies against Omicron induced by the third dose of inactivated or mRNA vaccines could not be further enhanced by the fourth dose of vaccine. However, these studies found that the fourth dose was able to bring neutralizing antibody levels back to the peak after the third dose 79, 80, a phenomenon known as 'ceiling of immunity' 81. These results favor the idea of continuing routine vaccination as long as SARS-CoV-2 persistence.

## 4. Vaccine-induced T cell immune responses against Omicron variant

T cell-mediated immune responses constitute the other essential arm of immune defense in the control of intracellular pathogens. For example, it had been shown that T cell responses play a critical role in virus clearance in the infection of influenza viruses, SARS-CoV and MERS-CoV 82-84. In SARS-CoV-2 infection, lower frequencies of CD8 and CD4 T cells was associated with more severe disease 85, 86. A recent study indicated that the coordinated SARS-CoV-2-specific adaptive immune responses are mediated by CD4 and CD8 T cells, and both subsets of T cells are protective and associated with milder disease 87. CD4 and CD8 T cells are highly proliferative in patients infected with SARS-CoV-2 88, and both these replicating CD8 and CD4 T cells expressed the elevated levels of cytotoxicity markers 89. However, the effector function of T cells appears to fail in the severe patients 90. In patients with COVID-19, T cells underwent an extensive remodeling in terms of abundance and phenotype 91. The peak levels of SARS-CoV-2-specific memory CD4 and CD8 T cells was found to be in the first month of infection, and then slowly declined in the following 6~7 months. Central memory Th1 (T helper 1 cells) CD4 T cells dominate throughout the early infection and recovery period 92. By comparison with health individuals, Th1 cells present in COVID-19 patients characteristically expressed CXCR3 and CCR6, as well as transcripts encoding anti-viral cytokines and chemokines, including CD154, IFN-γ, IL-2 and TNF 92, while their expression of IFN-γ, IL-2 was lower than influenza-reactive Th1 cells 93. CD4 cells with cytotoxic activity, namely CD4 CTLs, can directly induce the death of major histocompatibility complex class II-expressing infected cells and recruit innate immune cells to enhance CD8 CTL responses 93. The proportion of SARS-CoV-2-reactive regulatory T cells (Treg) also significantly is reduced in hospitalized patients and patients with an impaired Treg response to SARS-CoV-2 have a more robust CD4 CTL responses 93 (reviewed by us recently 94). The levels of SARS-CoV-2-specific memory CD4 T cells are constant from 3 to 6 months following mRNA vaccines vaccination 39. The most of vaccinees are able to elicit a robust CD4 T cell response up to 6 months after vaccination and the early CD4 T cell responses and humoral immune responses are correlated 39. Robust CD8 T cells are crucial for the clearance of infected cells in the early phase of infection 90. A preclinical study on rhesus macaques showed that CD8 T cells could contribute to the protection against SARS-CoV-2 when humoral immunity is insufficient 95. During and after infection, GZMB (Granzyme B)-expressing CD8 T cells were clonally expanded, resulting in the generation of protective memory T cell responses against SARS-CoV-2 91. It was shown that memory CD4 T cells recognize SARS-CoV-2 virus structural and accessory proteins, while memory CD8 T cells prefer to recognize nucleocapsid protein 92. Also, these T cell responses last for at least 6 months, as revealed by an 8-month-long serological measurement at a population scale 41. All these evidence indicate that the robust T cell responses with the proper specificity, phenotype, and function induced by vaccines against SARS-CoV-2 can control the infection and time course of the disease, as well as can promote the optimization of the humoral immunity, although the major goal of most current vaccines is to elicit neutralizing antibodies 96, 97. It is proposed that an ideal COVID-19 vaccine should stimulate the production of neutralizing antibodies and induce robust T cell-mediated immune responses simultaneously. Previously, S-protein as antigen was focused on the identification of the most of vaccine candidates. Non-spike proteins may need to be included in the development of future vaccines, in order to induce a broad epitope coverage, a process akin to natural infection of virus 97.

It is interesting to ask if the mutation of SARS-CoV-2 would result in the evasion of T cell-mediated immune responses. Indeed, the single point mutation may eliminate the response of individual T cell clone. However, such mutation is unlike to abolish the overall cell-mediated immunity, for the reason that T cell receptors can recognize over 1,400 different SARS-CoV-2 epitopes (382 for CD4 cells and 1,052 for CD8 cells) at the population level 98. A study showed that 93% of CD4 and 97% of CD8 T cell epitopes in COVID-19 patients infected with different variants are completely conserved 99. And the SARS-COV-2-specific CD4 and CD8 T cell responses are less dominated by spike protein epitopes, and the mutation on spike protein will not influence the T cell responses 100. All these results suggest that SARS-CoV-2-specific T cell responses may remain robust cross-reactive to Omicron variant and protect vaccinees from severity and death, even though all vaccines were developed based on an ancestral virus.

Indeed, it was shown that SARS-CoV-2-specific T cell responses induced by current vaccines retain robust reactivity to Omicron variant, albeit such crucial role of T cell immunity is relatively underestimated 101. In fact, the response of polyclonal memory T cells is not affected by single amino acid substitutions or deletions across large peptidomes 99. In a study found that the individuals with primary series vaccination with mRNA1273, or BNT162b2, or Ad26.COV2.S have similar median effector T cell reactivity (in terms of IFN-γ ELISpot spot forming units of SFU/10^6^ PBMCs) against Omicron S-protein, as compared with that against WT SARS-CoV-2 (WT: 43, Omicron: 42), while prior infection could bring better performance (WT:311, Omicron:315 ). Even in individuals with undetectable Omicron neutralizing antibody, effector T cell responses were still measurable 102. Based on samples obtained at 28 days after vaccination with ChAdOx-1, or Ad26.COV2.S, or mRNA-1273, or BNT162b2, a study showed that 94% individuals (46/49 ) had S-specific CD4 T cells and there were no significant differences between the responses of these CD4 T cells to WT, Omicron and other VOCs 103. In contrast, the responses of CD8 T cells to Omicron could only be detected in 63% of recipients (31/49). These T cells responses were maintained at least for 6 months after booster dose 103. Also, mRNA booster vaccine recipients had significantly stronger T cells responses to Omicron S-protein at the time of breakthrough infection 104. In another study, it was found that about 15% vaccinated individuals (5 out of 32) exhibited a loss of CD8 T cell recognition of Omicron 105. This study also showed that the vaccination with 2-dose of BNT162b2 or 2-dose of Ad26.COV2.S could trigger CD4 T cell responses to the Omicron 105, while Ad26.COV2.S induced stronger spike-specific CD8 T cell responses 106. At 8 months after vaccination, the median CD8 T cell responses were 0.016% with the BNT162b2, 0.017% with the mRNA-1273, and Ad26.COV2.S were highest among them which was 0.12% 107. It was shown that the vaccination with BNT162b2 or Ad26.COV2.S could elicit broadly CD4 and CD8 T cell responses against Omicron variant, including in the subsets of central and effector memory cells, and such T cell responses were durable and could retain 8 months after vaccination 108. In the macaques vaccinated with BNT162b2 and Ad26.COV2.S, 4 of 30 failed to control viral replication with negligible Omicron-specific CD8 T cell response even they had moderate neutralizing antibodies titers, other 26 macaques demonstrated rapid control of virus 109. It is worth mentioning that, similar to the humoral responses of the heterologous vaccination regimens, the advantage of a diverse immune response has been demonstrated in cell-mediated immunity. For example, one study found that individuals vaccinated with BNT162b2 largely preserved CD8 T cell recognition of Omicron S-protein epitopes because about 85.3% (n = 208) of the HLA class I epitopes were not affected on the amino acid sequence level 46. Another study reported that SARS-CoV-2 spike-specific CD4 and CD8 T cells in individuals vaccinated with BNT162b2 provided extensive immune protection against Omicron, with median frequencies of antigen specific CD4 T cells and CD8 T cells that cross-recognized Omicron were 91% and 92%, respectively 110. The peripheral blood mononuclear cells from 8 donors vaccinated with BNT162b2 demonstrated equivalent levels of responses to both ancestral and Omicron S-protein 111. Tracking individual clones of SARS-CoV-2-specific CD8 T cells showed that an IFN signature marked long-lived, circulating memory CD8 T cells persisting even 1-year, and such cells expressed CD45RA, IL-7 receptor-α and T cell factor 1 112. Thus, T cell responses elicited by vaccines against Omicron can maintain for a considerable long time.

The performance of inactivated vaccine CoronaVac is poorer than mRNA vaccines in eliciting the generation of neutralizing antibody. However, inactivated vaccines appeared to have the capacity to induce even stronger CD4 and CD8 T cell responses to the structural protein, such as highly abundant, conserved, and immunogenic N protein, as compared with BNT162b2 113. This result is in line with the results of two clinical studies in Turkey and Chile which showed that inactivated vaccines effectively prevented COVID-19, including severe disease and death 114, 115. CoronaVac could induce more effector memory T cell response to S, N, Envelope and Matrix protein of SARS-CoV-2, yielding long-term cross-reactive cellular memory during infection with SARS-CoV-2 variants and the average magnitude of structural and S-specific T-cell responses were higher in CoronaVac-vaccinated subjects than BNT162b2-vaccinated subjects 113. Theoretically, more broad cellular immune responses can be triggered by whole viral protein contained in inactivated vaccine. In recipients of inactivated vaccine, T cell epitope recognition is abundant and the most of them are conserved 98, while Omicron variant have not evolved extensive mutation which can escape T cell responses because of the variety of HLA haplotypes between individuals and virtually all individuals with existing SARS-CoV-2-specific CD8 T cells recognize Omicron variant 116. This may explain why current inactivated vaccine (CoronaVac) rendered same effectiveness in protection against severe outcome of patients with Omicron variant, as compared to BNT162b2 117, although inactivated vaccines is known to elicit weaker humoral immune responses against Omicron. Moreover, it was shown that CoronaVac recipients had higher levels of FcγRIIIa-binding antibodies that could stimulate antibody-dependent cell cytotoxicity of NK cells, as compared with that in BNT162b2 recipients 113.

T cell responses against non-spike structural and accessory proteins of WT and Omicron variants are preserved in all individuals with prior infection, while individuals only received S-protein-targeting vaccine without prior infection lack of such responses 102. A study showed that effector T cells increase more in individuals boosted with BNT162b2, as compared to individuals boosted with CoronaVac, in those received CoronaVac as prime vaccination 118. These support the notion that vaccination with vaccines using different technical platforms which target various epitopes can elicit a wider range of T cell responses, and repeated exposure to antigen may enhance cross-reactive T cell responses. Moreover, using different vaccine regimens in prime and booster vaccinations may represent a better solution for emerging variants. In particular, the use of inactivated vaccines containing whole virus proteins which is rich in highly conserved and immunogenic proteins to stimulate T cell responses, may provide better protection. To date, the understanding of responses of T cells to Omicron remains incomplete. Therefore, in-depth understandings of the molecular basis underlying T cell responses which play a decisive role in the resistance of transmission and infection of Omicron variant need further investigation.

## 5. Closing remarks

To date, all the current vaccines are designed based on the ancestral SARS-CoV-2 virus, thus there is an urgent need for the new generation of vaccines specifically targeting Omicron variant or its subvariants (such as BA.2, BA.3, Deltacron and XE). In fact, it is the current efforts of pharmaceutical companies including Pfizer and BioNTech^119^. However, there are some potential challenges for the development of Omicron vaccines. For example, antigenic and virological characterization of the various Omicron sub-variant BA.1, BA.2, BA.3, Deltacron and XE remain to be thoroughly studied, while the new mutant strains may emerge before Omicron vaccines can be produced and delivered on a large scale. Since the performance of any newly developed vaccine may be affected by acquired immunity triggered by previous vaccines, further research is also needed to address questions such as whether different vaccines interact with each other, and if epitope similarities or differences between sub-variants affect the performance of Omicron vaccines. Nevertheless, further understanding of humoral and cellular immune responses, as well as their underlying molecular mechanism, elicited by current vaccine regimens against Omicron variant would be crucial for containing current surge of COVID-19 with existing tools. Moreover, in-depth study in this line could also be helpful to device future vaccines against emerging mutant strains of SARS-CoV-2. To develop vaccines constantly against new SARS-CoV-2 variants does not seem to be a long-term solution, especially for a rapidly mutated virus like SARS-CoV-2. More innovative technologies should be developed. For example, it was proposed that a cocktail vaccine that combines the structural and non-structural viral antigen for a broader range immunity 120, or contains the protein that is highly conservative from virus such as nucleocapsid structural protein 121, 122 may effective in protection against emerging variants.

Taken together, recent studies have been focusing on the understanding of humoral as well as cellular immune responses against Omicron variants elicited by current vaccine regimens, which underlies the effectiveness of vaccines on the highly mutated variants of SARS-CoV-2. These studies clearly indicate that the levels of vaccine-elicited neutralizing antibody against Omicron variants are reduced to various degrees depending on the route of technology, while robust T cell mediated immune responses are largely preserved in the individual who has received any approved vaccines. This at least partially explains why the infectivity and transmissibility of SARS-CoV-2 Omicron variant is extremely high, while the clinical severity and death are markedly reduced. There is compelling evidence that the booster dose as well as heterologous vaccination represent a strategy to enhance the overall protective efficacy of current vaccines. Further understanding of cellular and molecular basis underlying the Omicron-specific humoral as well as cellular immune responses elicited by current vaccines would be helpful for the design of more effective vaccines to prevent the emerging variants of SARS-CoV-2.

## Figures and Tables

**Table 1:** WHO emergency use listing

WHO EUL holders	Name of Vaccine	Vaccine type
BioNTech	BNT162b2	mRNA
Moderna Biotech	mRNA-1273	mRNA
AstraZeneca	ChAdOx1(AZD1222)	adenoviral vector
Serum Institute of India	Covishield	adenoviral vector
Janssen/Johnson & Johnson	Ad26.COV2.S	adenoviral vector
Sinopharm / BIBP	BBIBP-CorV	Inactivated
Sinovac Life Sciences	Coronavac	Inactivated
Bharat Biotech	COVAXIN	Inactivated
Serum Institute of India	Covovax	Recombinant subunit
Novavax	Nuvaxovid	Recombinant subunit

The vaccine approved by WHO listed in EUL to expediting the availability of these products to people affected by a public health emergency.

**Table 2:** The protective efficacy of 2 doses various vaccines against Omicron.

Vaccine type	Vaccines		GMT (NT50)	Effective(fold)	Compared to	Reference
mRNA vaccines	BNT162b2/mRNA-1273	BNT162b2/mRNA-1273	121	-22.9x	D614G	51
	BNT162b2/mRNA-1273	BNT162b2/mRNA-1273	17	-30.6x	USA-WA1/2020	52
	BNT162b2	BNT162b2	1.71(OD)	-1.4x	WT	74
	BNT162b2	BNT162b2		-23x	USA-WA1/2020	44
	BNT162b2	BNT162b2	22	-25.5x	D614G	45
	BNT162b2	BNT162b2	54	-29.8x	Victoria	50
	BNT162b2	BNT162b2	5.43-6.32	-35.7∼39.9x	WT	43
	BNT162b2	BNT162b2	7	-22.9x	WT	46
	BNT162b2	BNT162b2		-122x	WT	42
	BNT162b2	BNT162b2	1.11	-14.91x	WT	47
	BNT162b2	BNT162b2	89	-22x	WT	123
	BNT162b2	BNT162b2		-16x	D614G	48
	BNT162b2	BNT162b2	7	-30.9x	WT	57
	BNT162b2	BNT162b2	9 (ID50)	-14.3x	WT	49
	BNT162b2	BNT162b2	6%(EC50>30)	-16.6x	D614G	68
	BNT162b2	BNT162b2	9/20(IC50>16)	-2.2x	Alpha	54
	mRNA-1273	mRNA-1273		-43x	WT	42
	mRNA-1273	mRNA-1273		-42x	USA-WA1/2020	44
	mRNA-1273	mRNA-1273	27	-84x	D614G	70
	mRNA-1273	mRNA-1273	62	-49x		
	mRNA-1273	mRNA-1273	1/10(IC50>16)	-9x	Alpha	54
Adenovirus vector vaccines	Ad26.COV2.S			-17x	WT	42
	ChAdOx1	ChAdOx1	1.17(OD)	-2.0x	WT	74
	ChAdOx1	ChAdOx1	10	-13.3x	Victoria	50
	ChAdOx1	ChAdOx1	0/10(IC50>16)	-10x	Alpha	54
	ChAdOx1	BNT162b2	14/20(IC50>16)	-1.4x		
	Covishield	Covishield		-23.15x	B.1	55
	ChAdOx1	ChAdOx1	11%(EC50>30)	-8.1x	D615G	68
Inactivated vaccines	BBIBP-CorV	BBIBP-CorV	9.63	-7x	WT	60
	BBIBP-CorV	BBIBP-CorV	55	-1.52x	WT	61
	CoronaVac	CoronaVac	Inactive		USA-WA1/2020	56
	CoronaVac	CoronaVac	5	-6.4x	WT	57
	CoronaVac	CoronaVac	20.9			59
	CoronaVac	CoronaVac	53	-12.5x	WT	58
	COVAXIN	COVAXIN	4.9	-12.49x	B.1	63
	COVAXIN	COVAXIN	4.11	-8.67x	B.1	62

If not specifically marked, the GMT(NT50) represented geometric mean of 50% neutralizing antibodies titers. EC50 represented 50% effective concentration. IC50 represented 50% inhibitor concentration. ID50 represented inhibitory serum dilution. Due to different studies use some different standard. OD represented optical density. OR represented odds ratio (positive/negative). USA-WA1/2020 represented the strain isolated from the first patient in the United States. We make the results more uniform by using multiples of the data of Omicron and others (WT virus or other variants).

**Table 3:** The protective efficacy of booster doses various vaccines against Omicron.

Vaccine regimen	Prime vaccination		Booster dose	GMT	Effective (fold)	compared to	Reference
Homologous vaccination	BNT162b2/mRNA-1273	BNT162b2/mRNA-1273	BNT162b2/mRNA-1273	3179	+26.3	2-dose	51
	BNT162b2/mRNA-1273	BNT162b2/mRNA-1273	BNT162b2/mRNA-1273	93	+5.4x	2-dose	52
	BNT162b2/mRNA-1273	BNT162b2/mRNA-1273	BNT162b2/mRNA-1273		+38x	2-dose	67
	BNT162b2	BNT162b2	BNT162b2	3.37 (OD)	+1.9	2-dose	74
	BNT162b2	BNT162b2	BNT162b2		-4x	D614G	48
	BNT162b2	BNT162b2	BNT162b2	700	+31.8	2-dose	124
	BNT162b2	BNT162b2	BNT162b2	164	+23.4	2-dose	46
	BNT162b2	BNT162b2	BNT162b2		+27x	2-dose	42
	BNT162b2	BNT162b2	BNT162b2	114.9	+16x	2-dose	57
	BNT162b2	BNT162b2	BNT162b2		-7.5	USA-WA1/2020	44
	BNT162b2	BNT162b2	BNT162b2	107.6	+96.9x	2-dose	47
	BNT162b2	BNT162b2	BNT162b2	100%(EC50>30)	+16.7x	2-dose	68
	BNT162b2	BNT162b2	BNT162b2	1195 (ID50)	+132.8x	2-dose	49
	mRNA-1273	mRNA-1273	mRNA-1273		+19x	2-dose USA-WA1/2020	42 44
	mRNA-1273	mRNA-1273	mRNA-1273		-16.7		
	mRNA-1273	mRNA-1273	mRNA-1273	620	+24.1x	2-dose	70
	mRNA-1273	mRNA-1273	mRNA-1273	2002	+32.3x		
	BBIBP-CorV	BBIBP-CorV	BBIBP-CorV	48.73	+5.1x	2-dose	60
	BBIBP-CorV	BBIBP-CorV	BBIBP-CorV	84	+1.5x	2-dose	61
	BBIBP-CorV	BBIBP-CorV	BBIBP-CorV	22.96	-16.07x	WT	72
	CoronaVac	CoronaVac	CoronaVac	7.6	+1.5x	2-dose	57
	COVAXIN	COVAXIN	COVAXIN	76.14	+18.53x	2-dose	62
Heterologous vaccination	Ad26.COV2.S		mRNA-1273		+4x	1-dose Ad26.COV2.S	42
	ChAdOx1	ChAdOx1	BNT162b2	3.46 (OD)	+2.9x	2-dose ChAdOx1	74
	Covishield	COVAXIN			+1.65x	2-dose Covishield	55
	BBIBP-CorV	BBIBP-CorV	ZF2001	95.86	+10x	2-dose BBIBP-CorV	60
	BBIBP-CorV	BBIBP-CorV	ZF2001	172	+3.1x	2-dose BBIBP-CorV	61
	CoronaVac	CoronaVac	BNT162b2		+10.1x	2-dose CoronaVac	56
	CoronaVac	CoronaVac	BNT162b2	52.8	+10.6x	2-dose CoronaVac	57
	CoronaVac	CoronaVac	BNT162b2	575.8	+27.5x	2-dose CoronaVac	59

If not specifically marked, the GMT(NT50) represented geometric mean of 50% neutralizing antibodies titers. EC50 represented 50% effective concentration. IC50 represented 50% inhibitor concentration. ID50 represented inhibitory serum dilution. Due to different studies use some different standard. OD represented optical density. OR represented odds ratio (positive/negative). USA-WA1/2020 represented the strain isolated from the first patient in the United States. We make the results more uniform by using multiples of the data of Omicron and others (WT virus or other variants) and the result of booster dose was compared with 2-dose.

## Abbreviations

SARS-CoV-2: Severe Acute Respiratory Syndrome Coronavirus 2
RBD: receptor binding district
EUL: emergency use listing
VOC: variants of concern
NTD: N-terminal structural domain
GMT: geometry mean titers
Th1: T helper 1 cells
Tfh: follicular helper T cell
TNF: Tumor necrosis factor
IFN-γ: Interferon-γ
WT: Wild type
ACE2: angiotensin-converting enzyme II
S-protein: Spike-protein
GMNT: geometry mean neutralization titers
